# Single-station seismic microzonation using 6C measurements

**DOI:** 10.1007/s10950-020-09944-1

**Published:** 2020-08-05

**Authors:** Sabrina Keil, Joachim Wassermann, Heiner Igel

**Affiliations:** Department of Earth and Environmental Sciences, LMU Munich, Theresienstraße 41, 80333 Munich, Germany

**Keywords:** Microzonation, Rotational seismology, Ambient noise

## Abstract

Microzonation is one of the essential tools in seismology to mitigate earthquake damage by estimating the near-surface velocity structure and developing land usage plans and intelligent building design. The number of microzonation studies increased in the last few years as induced seismicity becomes more relevant, even in low-risk areas. While of vital importance, especially in densely populated cities, most of the traditional techniques suffer from different shortcomings. The microzonation technique presented here tries to reduce the existing ambiguity of the inversion results by the combination of single-station six-component (6C) measurements, including three translational and three rotational motions, and more traditional H/V techniques. By applying this new technique to a microzonation study in the downtown area of Munich (Germany) using an iXblue blueSeis-3A rotational motion sensor together with a Nanometrics Trillium Compact seismometer, we were able to estimate Love and Rayleigh wave dispersion curves. These curves together with H/V spectral ratios are then inverted to obtain P- and S-wave velocity profiles of the upper 100 m. In addition, there is a good correlation between the estimated velocity models and borehole-derived lithology, indicating the potential of this single-station microzonation approach.

**Highlights**

- Testing a novel method for microzonation combining single-station six-component measurements and H/V ratios
- Estimation of Love and Rayleigh wave dispersion curves from single-station six-component data
- Positive correlation between inverted velocity profiles and borehole-derived lithology

## Introduction

In seismic microzonation, the velocity structure of the upper few 100 m is estimated in order to characterize the local earthquake shaking characteristics. There are two common methods that allow the determination of 1D subsurface wave velocity structures: (1) array-based methods, such as spatial autocorrelation (SPAC) (Aki 1957) and frequency-wavenumber (FK) analysis (Capon 1973); (2) single-station approaches, including horizontal-to-vertical (H/V) spectral ratios (Nogoshi and Igarashi 1971). In general, array-based methods are well understood and give reliable results (e.g., Marano and Fäh (2014)). However, as a severe limitation, the installation and maintenance in an urban area are very complex. Due to its simplicity, the single-station H/V method is commonly applied in microzonation studies, but its theoretical foundation is still not completely understood and the results highly depend on the quality of the noise (e.g., Malischewsky and Scherbaum (2004)).

Wassermann et al. (2016) demonstrated that a single-station six-component approach, combining rotational motion measurements (which are related to the gradient of a seismic wave field) with traditional translational recordings (i.e., recordings of ground velocity), may give comparable results to array techniques for the estimation of the 1D local velocity structure and the dominant direction of the incident wave field.

In addition, Bernauer et al. (2018) showed that a portable and reliable broadband rotational sensor, the blueSeis-3A (iXblue 2017), is now available. In order to test the performance of this six-component approach in a real-world application, we conduct an experiment using a Trillium Compact seismometer and the blueSeis-3A rotational sensor within Munich, where the largest inner city geothermal power plant gives rise to concerns about induced seismicity in a densely inhabited area. Using noise measurements, the relation between rotational and translational motion is exploited to estimate Love and Rayleigh wave dispersion curves, which can then be inverted for the local 1D velocity structure (Wassermann et al. 2016). To get as much information on the subsurface as possible, the H/V method is used to complement the data in the lower frequency range (< 5 Hz). Finally, the inverted velocity models are compared with lithologic profiles, derived during the GeoPot (Geo-potentials of the tertiary subsurface) project (TUM 2018) of the Technical University of Munich (TUM), to identify any correlations between wave velocity and geology.

## Data acquisition

The six-component measurements require the simultaneous recording of translational and rotational motion. In this study, two instruments are used; a Nanometrics Trillium Compact 120s seismometer and an iXblue blueSeis-3A rotational motion sensor (iXblue 2017). In order to record the same movement, the seismometer and the rotational motion instrument have to be installed on a single rigid base. Nevertheless, there also exist six-component instruments, so-called Rotaphones, which measure three rotational and three translational components in a fixed frame (Brokešová et al. 2012). The rotational seismometer blueSeis-3A (iXblue 2017) is based on an interferometric fiber-optic gyroscope (FOG), which is strictly insensitive to translational motions (Bernauer et al. 2018). A more detailed description of the working principle of FOGs is given in Lefevre (2014).

**Fig. 1:**
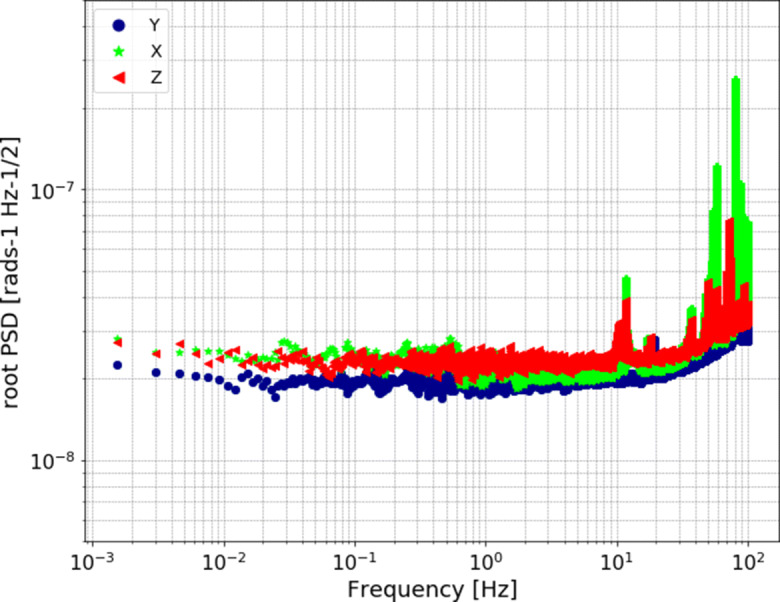
Self-noise power spectra of the three components of the blueSeis-3A (dots, triangles, and stars) determined during a laboratory experiment at the Geophysical Observatory in Fürstenfeldbruck, Germany. The sensor was placed on an auxiliary monument in a quiet location. All three components show flat self-noise levels over a frequency range of 0.001 to 50 Hz. The X-component exhibits significant peaks at 58 and 83 Hz, and the Z-component at 74 Hz, probably due to seismic ambient signals (cf. Bernauer et al. (2018))

Bernauer et al. (2018) conducted several laboratory tests in order to determine the performance of the blueSeis-3A. They found that the sensor has a flat self-noise level lower than 30 nrads^− 1^Hz^− 1/2^ over a wide frequency range of 0.001–50 Hz, as can be seen in Fig. 1. In addition, the sensor is very stable in changing ambient conditions, such as temperature and magnetic field, which makes it suitable for field installations (Bernauer et al. 2018).

**Fig. 2:**
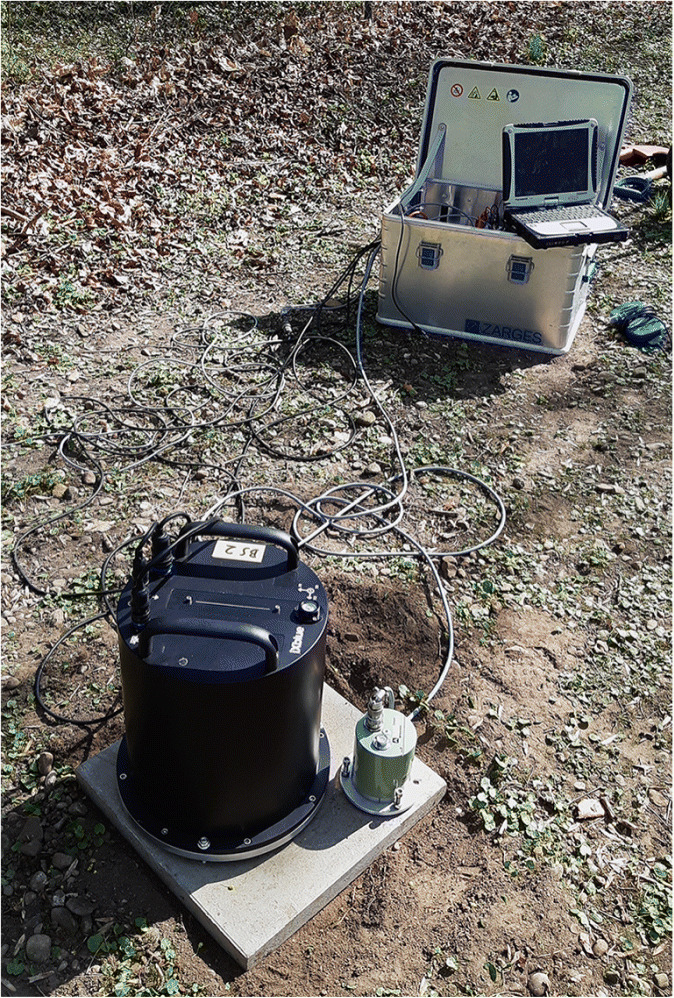
Setup for the single station six-component noise measurements. The two instruments, the blueSeis-3A rotational sensor on the left and the Trillium Compact 120s seismometer on the right, are placed about 10 cm apart on a concrete slab. The instruments are connected to GPS, power, and the seismometer to a REFTEK digitizer (inside of metal box). Before starting the recording of the data, a styrofoam insulation box is placed over the instruments

**Fig. 3:**
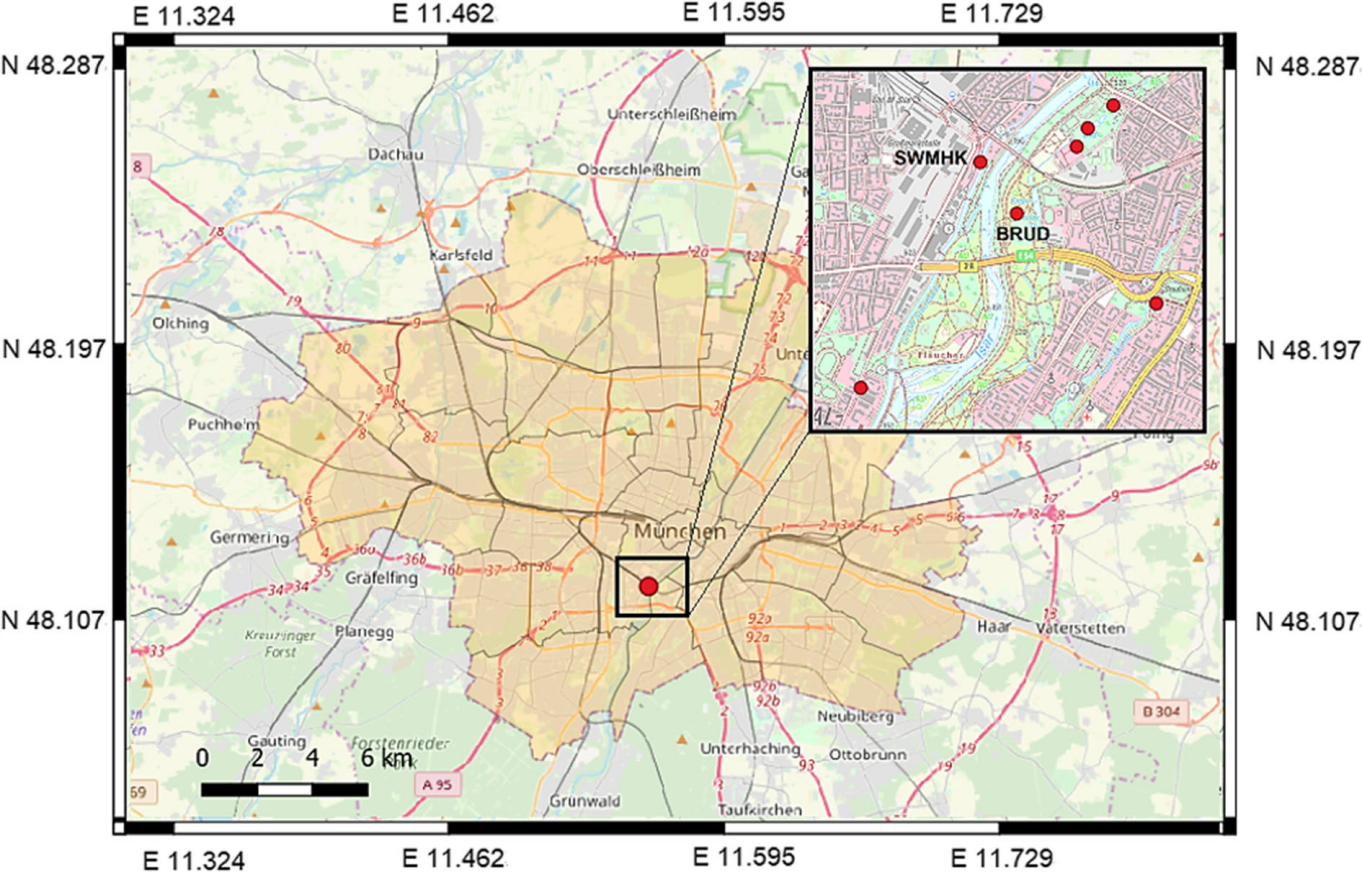
Map of Munich. The location of the geothermal power plant is marked by the dot. The study area is indicated by the rectangle. The magnified map in the upper right corner shows a part of the study area. The dots represent the measurement locations, including stations SWMHK and BRUD, which are discussed in more detail. Station SWMHK is located at the geothermal power plant

The instrument setup used in this study is shown in Fig. 2. For the installation of the Trillium Compact seismometer and the blueSeis-3A rotational sensor, a thin layer of the top soil has to be removed in order to establish better coupling to the ground. The two instruments are then placed next to each other on a concrete slab to guarantee a stable position and are connected to power and GPS. The seismometer is additionally connected to a REFTEK digitizer. The distance between the two instruments is about 10 cm, which is negligible compared with the smallest wavelength of a few meters. Therefore, the setup can be considered a single-station measurement, where both instruments record the same movement. To shield the devices against wind and direct sun radiation, a styrofoam insulation box is placed over them. Ambient noise is recorded during daytime with sampling rates of 200 Hz for approximately 2 h, which is enough to get a good representation of the wavefield in the recorded frequency range. In addition, increasing the measurement time to several days would make installation and maintenance more complicated and thus reduce the impact of the method. The desired frequency band for the estimation of the velocity profiles lies between 1 and 20 Hz, which corresponds to urban noise, as was shown by numerous authors (e.g., Asten (1984) and Gutenberg (1958)). Measurements are performed at several locations in the city of Munich in the vicinity of the landing points of the geothermal wells, since these are the most likely regions for the nucleation of induced earthquakes. The study area is marked in Fig. 3.

## Methods

### Love and Rayleigh wave dispersion estimation

Six-component measurements provide a new way of computing Love and Rayleigh wave dispersion curves. Under the premise that we only have to deal with plane, fundamental mode surface waves, it was shown by several authors (e.g., Ferreira and Igel (2009) and Kurrle et al. (2010)) that simple relations between the rotational motion and the translational acceleration of a seismic signal exist. In case of a fundamental mode Love wave, the relation is:

1 $$  c_{L}(f)= - \frac{a_{T}(f)}{2\dot{\omega}_{Z}(f)} $$

where *c*_*L*_(*f*) represents the frequency-dependent phase velocity, *a*_*T*_ the transversal component of acceleration, and $\dot {\omega _{Z}}$ the vertical rotation rate.

The transversal acceleration can be further defined as:

2 $$  a_{T}(f) = sin(\phi_{L})a_{N}(f) - cos(\phi_{L})a_{E}(f) $$

Where *ϕ*_*L*_ is the back azimuth of the wavefield, *a*_*N*_ the N–S component of acceleration, and *a*_*E*_ the E–W component, respectively. This allows Eq. 1 to be rewritten as:

3 $$  -2c_{L}(f)\dot{\omega}_{Z}(f){} ={} sin(\phi_{L})a_{N}(f) {}- {}cos(\phi_{L})a_{E}(f) $$

The phase velocity *c*_*L*_(*f*) and the back azimuth *ϕ*_*L*_ are both unknown properties, which have to be estimated in the following steps.

Accordingly, the relation for the phase velocity of fundamental mode Rayleigh waves can be derived by taking the ratio of the vertical component of acceleration *a*_*Z*_ and the transverse rotation rate $\dot {\omega }_{T}$ (e.g., Lin et al. (2011)).

4 $$  c_{R}(f) = \frac{a_{Z}(f)}{\dot{\omega}_{T}(f)} $$

By substituting $\dot {\omega }_{T}$ with its N–S and E–W rotation rate components ($\dot {\omega }_{N}$ and $\dot {\omega }_{E}$), Eq. 4 can be rewritten as:

5 $$  \frac{a_{Z}(f)}{c_{R}(f)} = sin(\phi_{R})\dot{\omega}_{N}(f) - cos(\phi_{R})\dot{\omega}_{E}(f) $$

To solve these equations and estimate the Love and Rayleigh wave dispersion curves, an updated version of the python package ROLODE (ROtational LOve Dispersion Estimation; Wassermann et al. (2016)) is used. This program simultaneously estimates the direction and the velocity employing the principle of orthogonal distance regression (ODR). To fulfill the additional assumption of a single source active at a time, the data are analyzed at each frequency band in short sliding time windows. Each time window gives an estimate of phase velocity and back azimuth. A kernel density estimation (kde) is used to bin the estimates in error weighted histograms and model these histograms with Gaussian functions. The quality of the fit can be improved by introducing a weighting scheme to the computation of the histograms, which accounts for the goodness of the straight line fit by the ODR (i.e., the correlation between the two quantities). The estimated phase velocities from the ODR are weighted according to:

6 $$  {w_{norm}^{x} = (1 - \frac{1}{w})^{x}} $$

where

7 $$  w = \frac{{\sum\nolimits_{i}^{I}} (\dot{\omega}_{Z}(f)[i])^{2}}{{\sum\nolimits_{i}^{i}}({\epsilon_{i}^{2}} + {\delta_{i}^{2}})} $$

with *I* the number of processed time windows, *x* > 0, and *δ* and *𝜖* the errors of the dependent and observed values in the ODR.

The mode of the kde function and its variance are then used to determine the phase velocity and the error. From the velocity estimates at each frequency band the dispersion curve is derived. The procedure is described in more detail in Wassermann et al. (2016).

### Horizontal-to-vertical spectral ratios

The method of horizontal-to-vertical spectral ratios (H/V) was first introduced by Nogoshi and Igarashi (1971), who described it as the ratio between the Fourier amplitude spectra of the horizontal and the vertical components of microtremors. Several more recent studies interpret the H/V ratio as the ellipticity *χ* of Rayleigh waves, which can be computed by dividing the horizontal over the vertical component of particle motion (e.g., Malischewsky and Scherbaum (2004)).

8 $$  \chi = \mid \frac{H}{V} \mid $$

Numerous authors (e.g., Sylvette et al. (2006) and Malischewsky and Scherbaum (2004)) have shown that the H/V ratio exhibits a pronounced peak close to the fundamental S-wave resonance frequency of the site, when the surface layer exhibits a sharp impedance contrast with the underlying stiffer formations. This makes the ellipticity an important parameter to reflect properties of the underground structure (Sylvette et al. 2006) and gives additional data, especially in the lower frequency band.

The H/V curves, computed with the Geophysical Signal Database for Noise Array Processing (GEOPSY) software package (Wathelet et al. 2004; Wathelet 2008), are used to complement the dispersion curves for the surface wave inversion. The 1D velocity profiles are then derived with the DINVER module in the GEOPSY software package, which implements a neighborhood algorithm (Wathelet 2008).

## Results and discussion

**Fig. 4:**
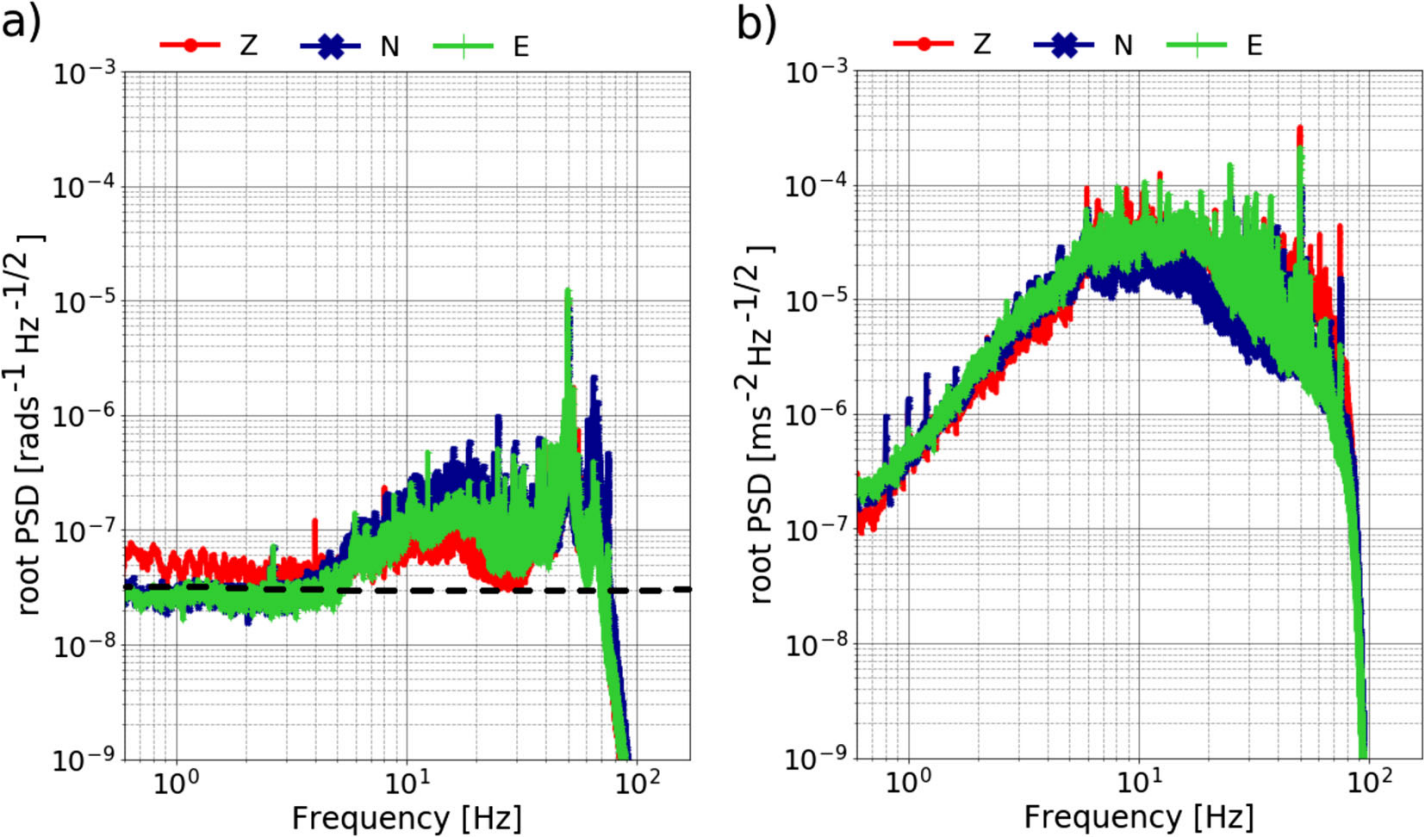
Power spectral density of the three components of the **a** blueSeis-3A rotational sensor and **b** Trillium Compact seismometer for station SWMHK. The dashed line in **a** marks the self-noise level of the rotational sensor, which lies at about 30nrads^− 1^Hz^− 1/2^

The power spectral densities (PSD) of the recorded rotational and translational data for station SWMHK, which is located right next to the geothermal power plant (Fig. 3) are presented in Fig. 4. It is easy to notice that the PSD for the translational components is about two orders of magnitude larger than for the rotational components. Additionally, an energy decrease toward lower frequencies can be observed in both cases. At about 5 Hz, the self-noise level of the rotational sensor is reached, which lies at 30nrads^− 1^Hz^− 1/2^ (Bernauer et al. 2018), explaining the flat trend of the curve in Fig. 4a.

**Fig. 5:**
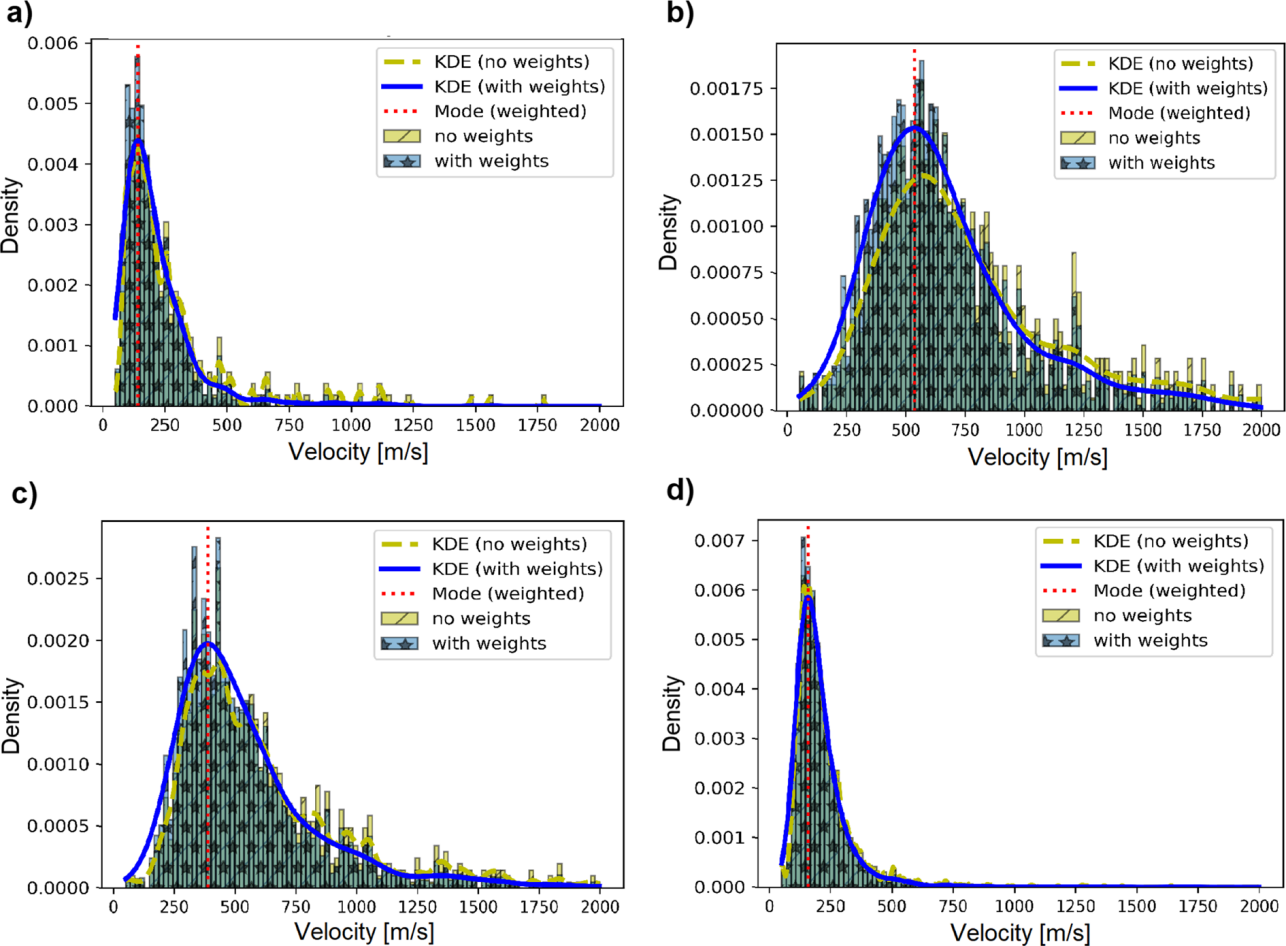
Histograms of estimated Love wave phase velocities using the SciPy ODR package for selected frequency bands: **a** 1.79 Hz, **b** 5.08 Hz, **c** 7.18 Hz, and **d** 14.35 Hz at station SWMHK. The bright bars represent the histogram of the non-weighted velocity estimates and the darker bars indicate the weighted estimates according to Eqs. 6 and 7. The dashed and solid curves give the best fitting Gaussian. The mode of the weighted distribution is shown as a vertical dotted line

**Fig. 6:**
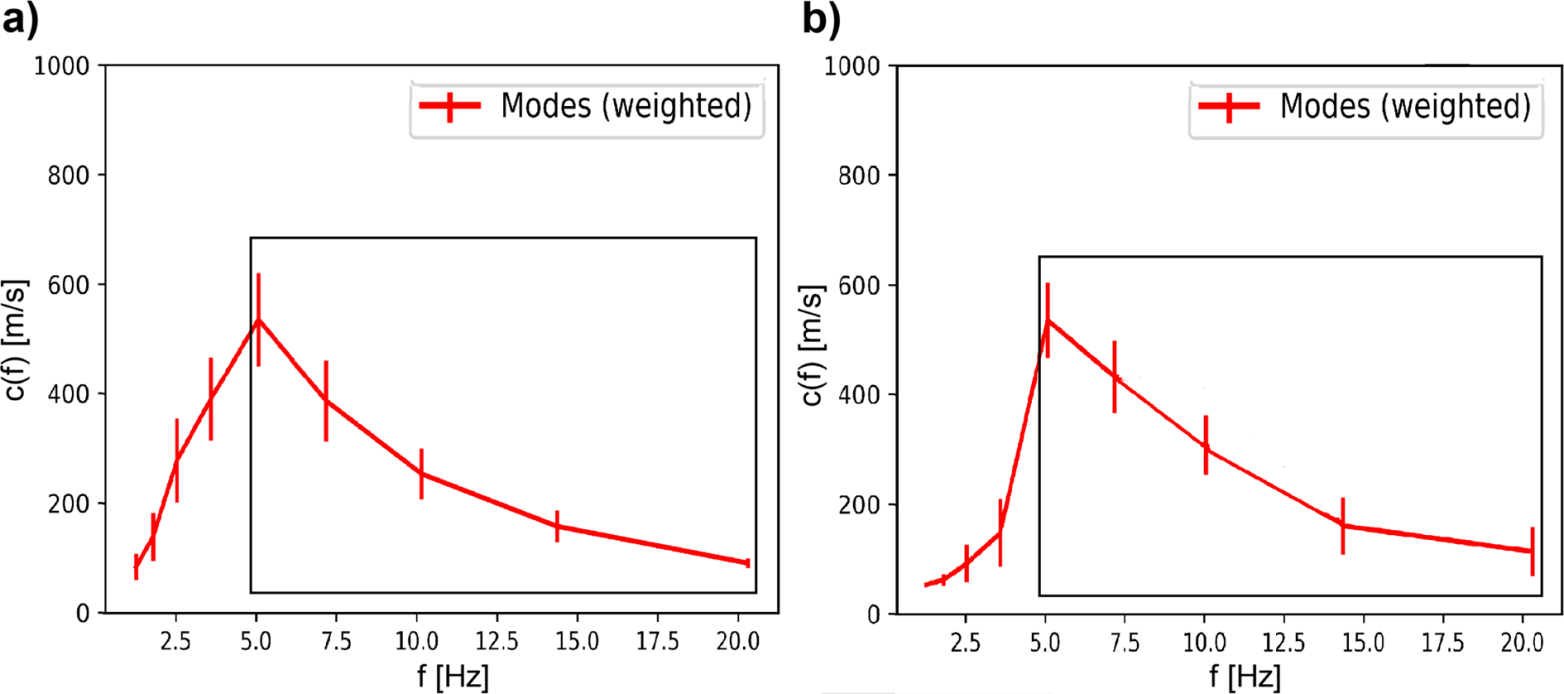
Estimated **a** Love and **b** Rayleigh wave dispersion curves using the ROLODE method. The error bars represent the standard deviations of each frequency band. The rectangular boxes give the frequency region where the inversion is applied

Applying the ROLODE method to the recorded data, the phase velocities for each frequency band can be estimated and are arranged in error weighted histograms. Figure 5 displays the distribution for four different frequencies. The data are modelled with Gaussian basis functions, where the mode is marked by a vertical dotted line. For each frequency band under consideration, using the mode as phase velocity estimate and the corresponding standard deviation of the kde function as the associated error, a complete dispersion curve can be derived. Figure 6 shows the resulting Love and Rayleigh wave dispersion curves for station SWMHK. Between 5 and 20 Hz, the data show a normally dispersive trend in which the phase velocity increases with decreasing frequency. However, below 5 Hz, the curves drop, indicating a limitation of this method in the lower frequency range. This is observed in the dispersion curves of all the measurements.

**Fig. 7:**
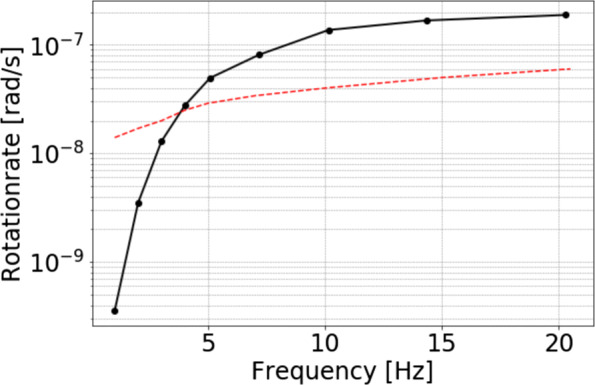
Expected rotation rates at station SWMHK calculated from a forward modelled Love wave dispersion curve and the measured translational data using (1). The dashed line marks the smallest rotation rates that can be recorded by the sensor. These values were extracted from an operating range diagram (ORD)

This limitation in the lower frequency range can be explained by re-visiting the PSD of the rotational sensor in Fig. 4a. At about 5 Hz, the self-noise level of the blueSeis-3A is reached and therefore no rotations can be recorded below that. This causes the drop in the estimated dispersion curves. Reasons could include either the lack of these lower frequencies in the noise spectrum of the city and/or the rotations are too small to be recorded by the rotational sensor. In this context, one has to keep in mind that the rotational motion amplitudes are equal to the ground acceleration values scaled by the ground velocity (cf. Eq. 1). Exploiting this relation, a rough estimate of the expected rotation rates can be calculated (Fig. 7). Therefore, a forward computation for the Love wave dispersion curve using the derived velocity profile at station SWMHK (Fig. 10) was performed. The phase velocity values together with the actual recorded translational data at this station were inserted into (1) to obtain the theoretical rotation rates. It can be seen that the rotation rates below 5 Hz are in the order of 10^− 8^ to 10^− 9^ and are therefore too small given the self-noise level of the sensor.

**Fig. 8:**
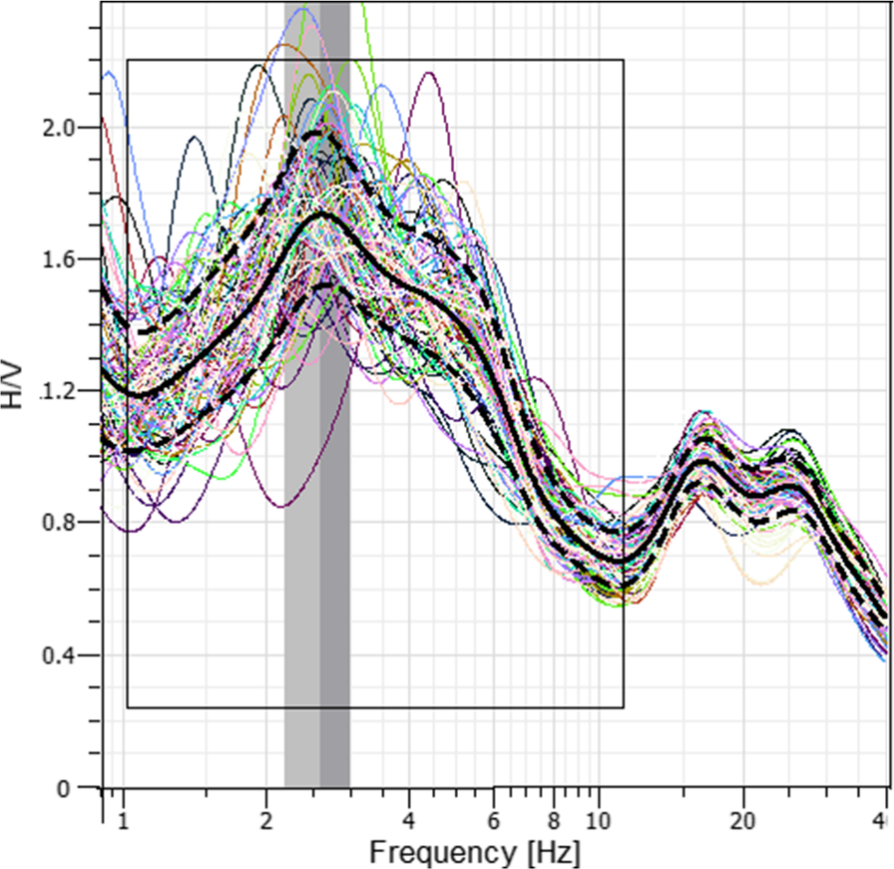
Ellipticity curve for station SWMHK computed with GEOPSY (Wathelet et al. 2004; Wathelet 2008). Each individual thin curve represents the computed H/V ratio for a single selected time window. The solid black curve indicates the geometrically averaged ellipticity curve over all individual H/V ratios. The two dashed lines represent the H/V standard deviation. The gray vertical bars mark the estimated ellipticity peak and its error. The rectangular box gives the frequency region where the inversion is applied

To complement the data in the lower frequency range and to enable an inversion to greater depth, the H/V curves are computed from the three translational components using the GEOPSY (Wathelet et al. 2004; Wathelet 2008) software package. The program divides the data into small time windows, for which the H/V ratio is computed separately. For the window selection, an anti-trigger algorithm is implemented with the objective to keep the most stationary parts of ambient vibrations and to avoid the transients, as explained in more detail by Bard et al. (2008). As a final step, an average over all single H/V ratios is computed. Figure 8 shows the ellipticity curve for station SWMHK, which exhibits a pronounced peak at about 2.5 Hz.

For the following joint inversion of a 1D ground velocity profile, the appropriate frequency range of the input data has to be selected, marked by the black boxes in Figs. 6 and 8. For the inversion at station SWMHK, the input consists of the H/V ellipticity estimates between 1 and 10 Hz, as well as the derived Love and Rayleigh wave dispersion curves. In case of the dispersion curves, normal dispersion is assumed, restricting us to use the data in the frequency range of 5–20 Hz. In the next step, the number of subsurface layers to be inverted for has to be defined. The neighborhood algorithm of the program then generates different ground models and computes the corresponding dispersion and H/V curves for each of those models. The comparison of the computation results with the measured dispersion and ellipticity curves provides a misfit value that indicates how far the generated model is from the data fit (Wathelet et al. 2004). In general, a velocity model with a low misfit value is desired; however, overfitting of the data has to be avoided. Increasing the number of parameters for the inversion most likely decreases the misfit because of the higher degree of freedom (DOF). Several tests with a different number of layers showed that the most significant reduction in the misfit can be achieved when increasing the number of model layers from 2 to 3. Because of that and in order to avoid overfitting of the data, a three-layer model for the inversion was chosen.

As part of the inversion, the linkage between the different free parameters (*v*_*p*_, *v*_*s*_, density, and Poisson’s ratio) has to be chosen. While Love waves consist of multiple reflected SH waves only, Rayleigh waves are a combination of P and SV waves. Because of that, the analysis of these surface waves provides more information about the S- rather than the P-wave. Therefore, the P-wave velocity model was linked to the S-wave model during the inversion by Poisson’s ratios between 0.2 and 0.5, which includes the typical values for soil and sedimentary rocks (Gercek 2007).

**Fig. 9:**
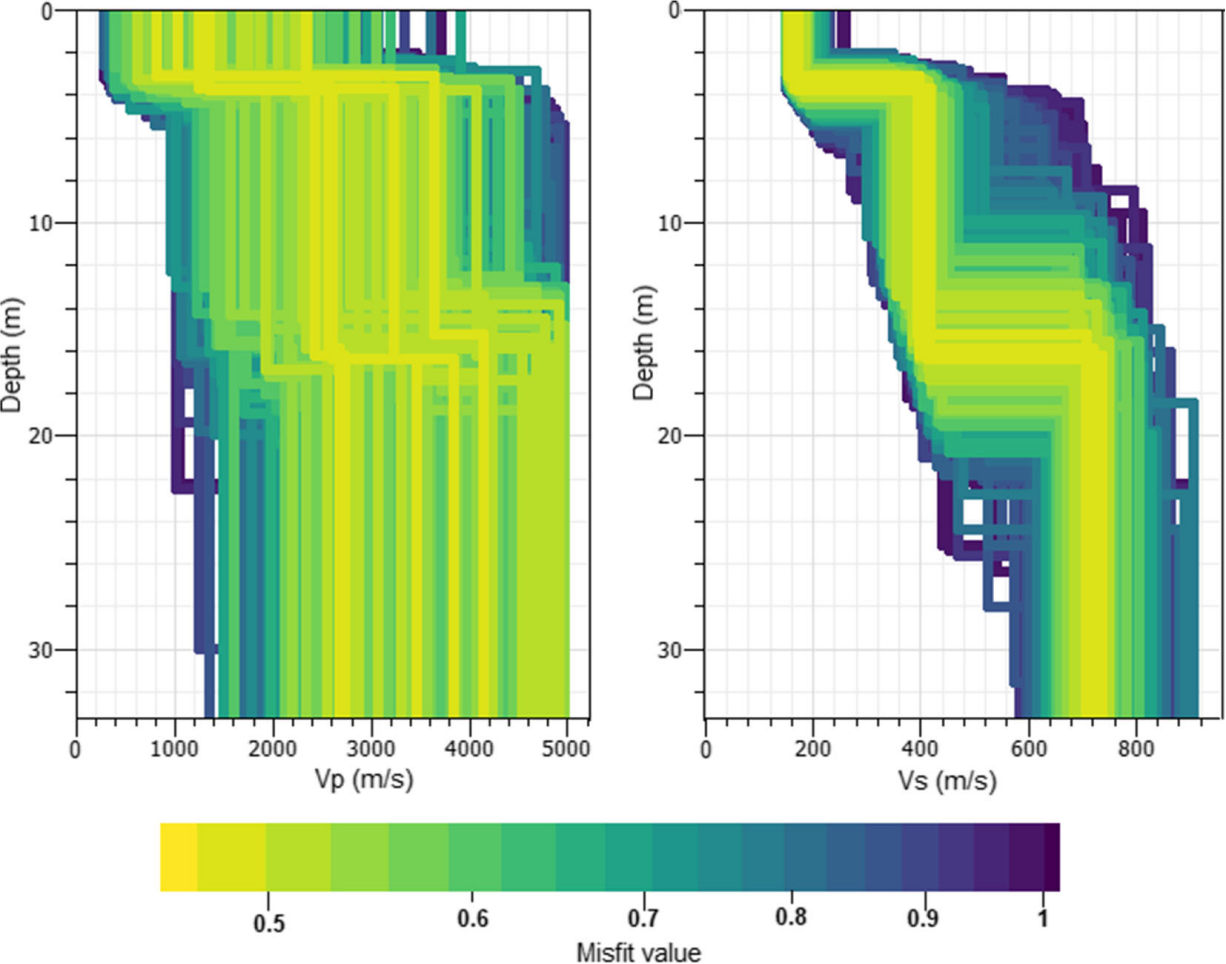
P- and S-wave velocity profiles for a three-layer inversion at station SWMHK using DINVER of GEOPSY (Wathelet 2008). Only Love and Rayleigh wave dispersion curves were used as an input. The shading gives the misfit of the computed models

The results for a three-layer inversion at station SWMHK, using only Love and Rayleigh wave dispersion curves as an input, are shown in Fig. 9. Due to the limitation of rotational motion in the lower frequency range, the inversion results are only sensitive to about 30 m depth. The velocity steps in the S-wave profile are well constrained, with the upper one at about 4 m depth and the second one at 14–16 m. Compared with this, the P-wave velocities are widely spread, even though the P-wave model was linked to the S-wave model. The reason for this is the large variance of the other free parameters due to the fact that there are less information on the P-wave.

**Fig. 10:**
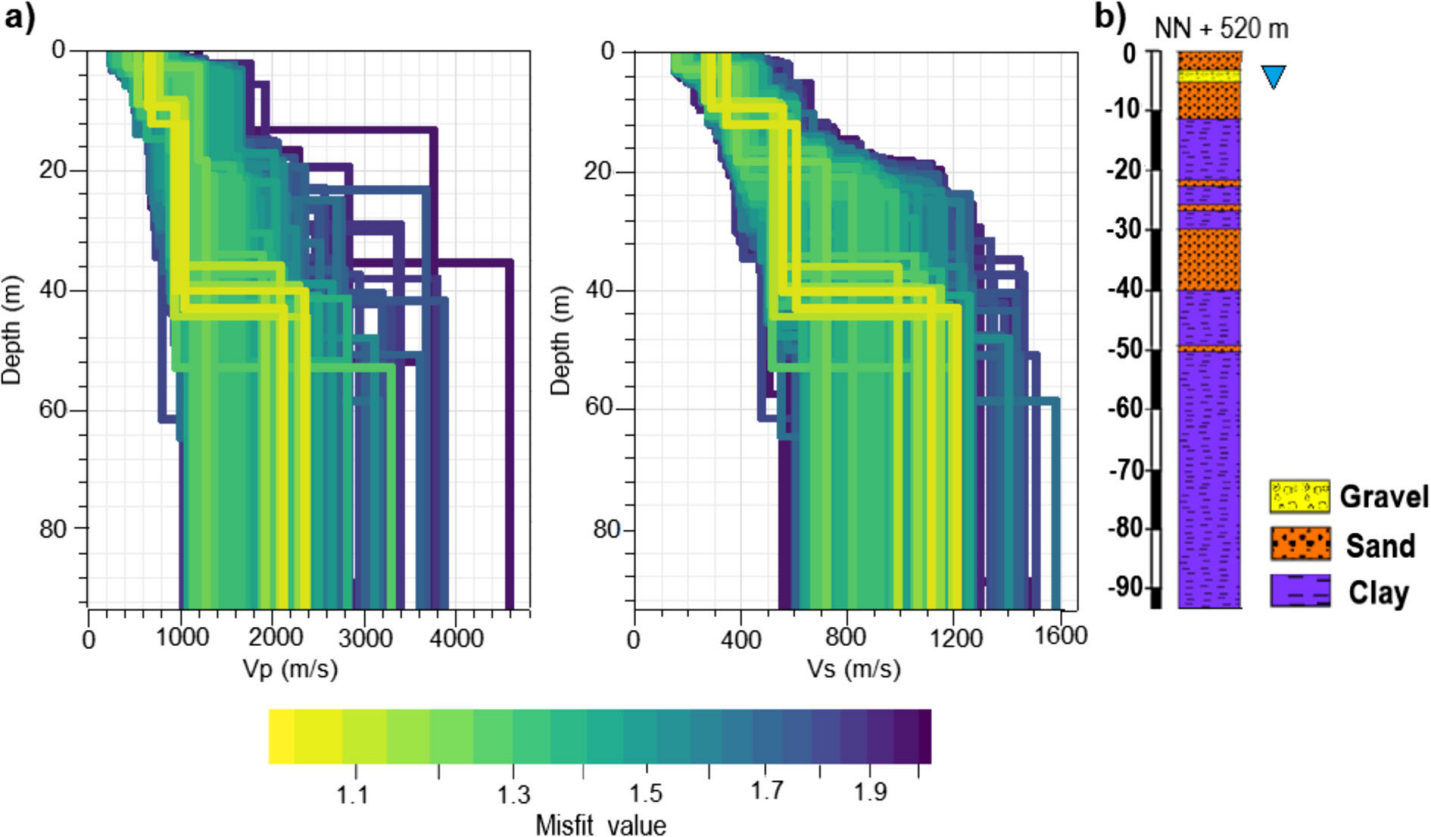
**a** Resulting three-layer P- and S-wave velocity profiles at station SWMHK using DINVER of GEOPSY (Wathelet 2008). For the inversion, Love and Rayleigh wave dispersion curves, together with H/V ratios, were used with a weight of 1. The shading gives the misfit of the computed models. **b** Lithologic profile estimated from borehole data during the GeoPot project of TUM (TUM 2018). The groundwater table is marked by the triangle

In order to get more information about deeper structures, the dispersion curves are complemented with the H/V ratios, which provide data to a lower frequency range. All input data are inverted with a weight of 1. Figure 10a gives the preferred P- and S-wave velocity model at station SWMHK for a three-layer inversion. There are two apparent velocity steps visible. Both velocity steps are shifted to greater depth, compared with the model in Fig. 9. Furthermore, the resolution of the P-wave profile increased due to the additional information from the Rayleigh wave ellipticity curve. Adding more layers to the inversion results in thin upper layers; however, the main velocity contrasts remain at the same depth range.

**Fig. 11:**
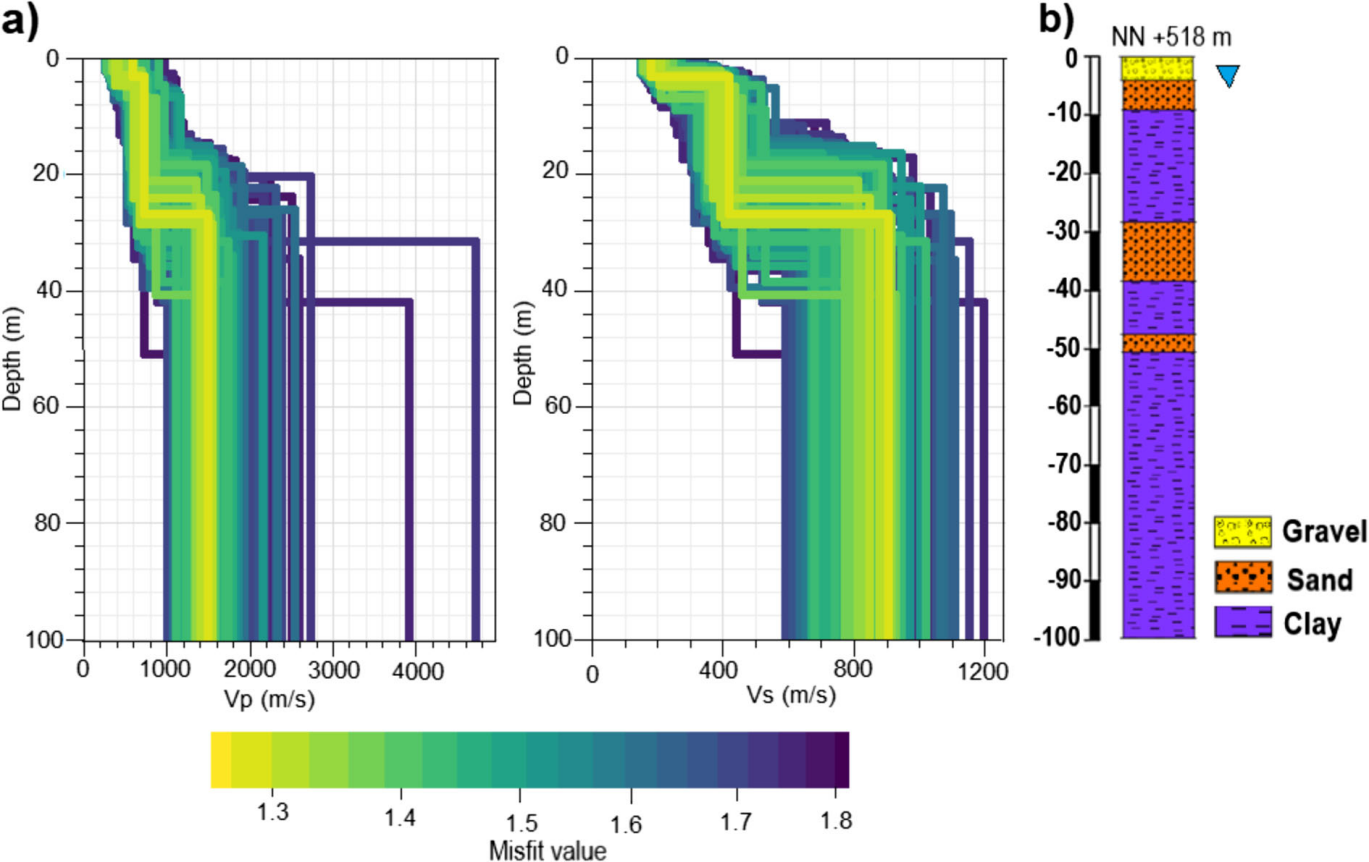
**a** Resulting three-layer P- and S-wave velocity profiles at station BRUD using DINVER of GEOPSY ((Wathelet 2008)). For the inversion Love and Rayleigh wave dispersion curves, together with H/V ratios, were used with a weight of 1. The shading gives the misfit of the computed models. **b** Lithologic profile estimated from borehole data during the GeoPot project of TUM (TUM 2018). The groundwater table is marked by the triangle

In the next step, the velocity profiles are compared with lithologic profiles to identify any correlation between the wave velocity and the geology. The lithologic profiles presented in Figs. 10b and 11b were extracted at the exact measurement locations from the geologic 3D model of Munich, derived during the GeoPot project (TUM 2018). The 3D model was constructed through interpolation of borehole data. Therefore, the lithologic profiles presented here are interpolated and could slightly deviate from the real structure. However, we assume that the deviation is small, since the borehole data are very dense within Munich. The distance between the closest boreholes and the stations SWMHK and BRUD is less than 100 m. Comparing the velocity profile of station SWMHK with the geology (Fig. 10), it is apparent that the upper velocity step at 8–10 m depth coincides with the change in lithology from sand to clay. In addition, the groundwater table also occurs at this depth range, which could influence the wave velocity. The second velocity contrast at 35–45 m depth coincides with a 10-m-thick sandstone layer. This indicates a correlation between the change in lithology and the increase in wave speed, while the very thin sandstone layers cannot be detected because of the decreasing resolution with depth.

Similar correlations can be found for the other stations. As a different example, the inversion results of station BRUD are shown in Fig. 11. Again, the P- and S-wave velocity profiles for the three-layer inversion compared with the 100 m deep lithologic profile estimated during the GeoPot project are displayed. The upper velocity increase occurs at 4–6 m depth, which coincides with the groundwater level at 5 m depth and the transition from gravel to sand. Also for location BRUD, as it is for station SWMHK, the velocity contrast might either reflect the presence of groundwater and/or the change in lithology. The second increase in velocity at 25–30 m depth lies in the range of the upper edge of the sandstone layer at 30 m depth. Therefore, the material change from clay to sand influences the wave speed. The sandstone lens at 50 m depth cannot be detected even when the number of layers in the inversion is increased because it is too thin for the resolution at this depth.

## Conclusion

The objective of this study was to test a new single-station technique for seismic microzonation in order to improve the resolution of the resulting 1D velocity models. The single-station approach using a Trillium Compact 120s seismometer and the blueSeis-3A rotational sensor makes measurements very simple in terms of logistics compared with an array setup, especially when working in an urban area. The six-component data allow the computation of Love and Rayleigh wave dispersion curves. The limitation of rotational motion in the lower frequency range (< 5 Hz), which appears to be a combination of sensor self noise and reduced rotational noise amplitudes, can be resolved by combining the dispersion curves with H/V ratios. The most reliable P- and S-wave velocity profiles are obtained by constraining the inversion to a three-layer model. Increasing the number of layers resolves more velocity steps close to the surface; however, the misfit value does not significantly decrease, which could be an indication for overfitting the data. As an application, the resulting 1D velocity profiles will be used in future studies to estimate the local shaking characteristics in Munich.

In general, the velocity models show a correlation to the lithologic profiles that were derived during the GeoPot project. Especially, layers close to the surface and the upper groundwater table could be identified.

The results have implications for any situations in which (1) near-surface velocity structures are sought and (2) multi-station networks are difficult or impossible to implement. This may happen in urban environments, at volcanoes, at the ocean bottom, or on planetary objects.
